# When Testing Becomes Learning—Underscoring the Relevance of Habituation to Improve Internal Validity of Common Neurocognitive Tests

**DOI:** 10.1111/ejn.70117

**Published:** 2025-04-24

**Authors:** Konstantin Warneke, Manuel Oraže, Gerit Plöschberger, Marco Herbsleb, Jose Afonso, Sebastian Wallot

**Affiliations:** ^1^ Institute of Psychology Leuphana University Lüneburg Lüneburg Germany; ^2^ Institute of Human Movement Science, Sport and Health University of Graz Graz Austria; ^3^ Viktor‐Frankl Hochschule, Pädagogische Hochschule Kärnten Klagenfurt am Wörthersee Austria; ^4^ Institute of Sport Science University of Klagenfurt Klagenfurt am Wörthersee Austria; ^5^ Department of Sports Medicine and Health Promotion Friedrich Schiller University Jena Jena Germany; ^6^ Department of Psychosomatic Medicine, University Hospital Jena Friedrich Schiller University Jena Jena Germany; ^7^ Centre of Research, Education, Innovation, and Intervention in Sport (CIFI2D), Faculty of Sport University of Porto Porto Portugal

**Keywords:** learning effects, Neurocognition, psychological measurement, repeatability, systematic testing error

## Abstract

Testing neurocognitive function is receiving growing attention in psychological and physical health research. To counteract the costs, reduced accessibility, and complexity of brain imaging (e.g., CT scans and fMRI) or function tests, neurocognitive performance tests (e.g., the Stroop test, the Trail Making Test, or the Choice Reaction Task) are commonly implemented. Although reliability is considered paramount when interpreting intervention effects, a detailed quantification of systematic and random errors is scarce. By recruiting 68 healthy participants from different age groups (7–64 years), we quantified population‐specific measurement errors in the aforementioned neurocognitive tasks. The goal was to raise awareness about the impact of learning effects on reliability assessments and their interpretation. By performing five testing sessions with two trials per day, we observed significant learning effects from repeated testing. Trial‐to‐trial improvements of up to 50% were measured, accompanied by a random measurement error reduction from day to day. These learning effects were task and population specific, highlighting the need for caution when transferring reliability coefficients from other studies. The quantification of systematic and random measurement errors underscores the importance of conducting sufficient habituation sessions in neurocognitive tasks, as test protocols lack validity if they do not ensure reliability. Therefore, sufficient habituation sessions (i.e., until no meaningful learning effects can be observed) may be warranted when testing is repeated within short timeframes.

## Graphical abstract

The graphical illustration shows the study design used to investigate interday and intraday reliability in common neurocognitive tasks. Performed over 5 days with two testing occasions per day, the course of 10 tests showed that up to four habituation sessions were necessary to minimize learning and habituation effects.

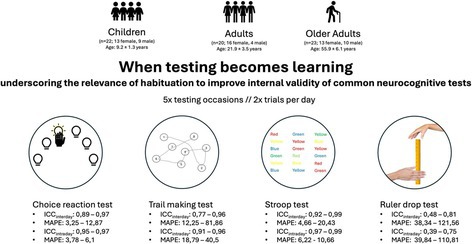

**Abbreviations**
ANOVA: analysis of variance
BA: Bland–Altman
ICC: intraclass correlation coefficient
M: mean
MAE: mean absolute error
MAPE: mean absolute percentage error
MDC: minimal detectable change
LoA: limits of agreement
SD: standard deviation
SEM: standard error of measurement

## 1 Introduction

Measuring cognitive function receives growing attention in both mental (Zorowitz and Niv 2023) and physical health research. Limited neurocognitive performance and concussion have been associated with proxies of brain injury and traumatic disorder rehabilitation (Allen et al. 2009; McAllister and McCrea 2017) such as stroke (Zanin et al. 2023), depression (Lerche et al. 2018), and gait parameters (stability and velocity) in the elderly (Hobert et al. 2011). The importance of neurocognitive performance measurement is not limited to elderly or patient populations: Recent literature has emphasized the relevance of including cognitive capacity‐enhancing programs in physical performance and injury prevention strategies for athletes (Wilke and Groneberg 2022; Wilke and Vogel 2020). In children, the determination of neurocognitive function focusses on educational settings as well as neurological and emotional pathologies, with emphasis on the importance of executive function and cognitive performance (Paine et al. 2021). Specifically, the literature suggests evaluating visual attention (Miller and Clapp 2011), inhibitory control (Periáñez et al. 2021; Scarpina and Tagini 2017; Stroop 1935; Takahashi and Grove 2020), and reaction time (Ferreira et al. 2024; Wilke and Vogel 2020). These examples underscore the relevance of neurocognitive assessment in health and performance sciences, which has contributed to the exponential increase in publications examining cognitive/executive function over recent decades (Alves et al. 2013).

Cognitive function includes lower and higher order functions or abilities. Wilke and Groneberg (2022) described lower order cognitive functions as primarily automatic baseline functions for higher order skills. Lower order functions include, but are not limited to, visual scanning, reaction time, processing speed, or short‐term memory and are often used interchangeably with executive function, which consists of three domains: working memory, inhibitory control, and cognitive flexibility. Brain function measurements via imaging techniques such as fMRI require heavy and expensive equipment. Therefore, brain adaptations in response to cognitive training are often assessed using neurocognitive tests (Wilke et al. 2020; Wilke and Vogel 2020). These tests are applied in various clinical settings to screen for mental pathologies related neurodegeneration (e.g., brain injury) (Allen et al. 2009) or depression (Lerche et al. 2018).

To implement neurocognitive testing batteries, a range of diagnostic tools has been developed that are described as valid and reliable for assessing both lower and higher order cognitive functions. For example, when aiming to explore working memory, inhibitory control, attention, or processing speed—functions that mix both lower and higher order abilities—psychological and neurocognitive research frequently applies the Stroop test (Faulkner et al. 2020; Faria et al. 2024; Wilke and Vogel 2020). Its subcategories, including word reading, color reading, and word‐color reading, were outlined to be reliable tools (intraclass correlation coefficient [ICC] = 0.89) (Galpin et al. 2008). Another easily applicable test for assessing processing speed, cognitive flexibility, and visuoperceptual tracking is the Trail Making Test (Llinàs‐Reglà et al. 2017; Suzuki et al. 2022; Tsiakiri et al. 2024). These are examples of neuropsychological measures for psychological health and cognitive performance measurements (Fossati et al. 2002). As described, neurocognitive function is also crucial in the context of physical health prevention, where the ability to react to external stimuli and initiate protective movements is frequently emphasized. This ability is commonly assessed using the Choice Reaction Task as a viable reflection of the individual's cognitive reactivity (Wilk et al. 2023; Wilke and Groneberg 2022) or the Ruler Drop Test as a potential, resource, and time‐saving alternative (Aranha et al. 2017).

The relevance of determining cognitive function is undisputed, but there is a need for improved reliability reporting and increased awareness about measurement errors. This is required to enhance the internal validity of the data. The reliability reporting issue focuses on two categories that are often neglected: the role of systematic and random measurement errors (Atkinson and Nevill 1998; Hopkins 2000). The ICC measures relative reliability and is a common method to justify a measurement protocol's reliability. Recently, Warneke et al. (2025) showed that ICCs classified as excellent can be accompanied by systematic learning effects and meaningful random errors (greater than 20%), raising questions about the validity of relying solely on ICCs. Furthermore, it is common to refer exclusively to ICCs from previous studies, assuming the transferability of reliability metrics between populations and contexts (Engeroff et al. 2019; Wilke et al. 2020; Wilke and Vogel 2020). This misinterpretation stems from a lack of awareness regarding protocol and test reliability. For instance, although the Stroop Test may be reliable overall, this does not mean that all participants can perform this test under consistent conditions, due to varying baseline values of inhibitory control and different learning and habituation rates.

In this study, we recruited participants from different age groups to explore whether there are age‐specific differences and variability in neurocognitive tasks (Bock and Girgenrath 2006; Bock and Schneider 2002) and to assess their influence on the reliability and measurement errors in frequently used neurocognitive tests. The Stroop Test, Trail Making Test, Ruler Drop Test, and Choice Reaction Task were selected due to their applicability across age groups. For a detailed measurement error and analysis of variance, both systematic (e.g., learning/fatiguing effects) and random errors (secondary variance, arising from unknown sources such as lack of standardization) were assessed.

## 2 Methods

To determine performance in a stable and consistent manner, several testing occasions conducted across days (interday) and within a single day (intraday) should yield the same outcome within a reasonable range (i.e., repeatability with reduced variation); at the same time, it should be reproducible between facilities, working groups and participants to ensure general reliability (repeatability and reproducibility) (Atkinson and Nevill 1998; Barnhart et al. 2007). To assess various facets of reliability for the listed neurocognitive tests, participants performed the Ruler Drop Test and the Choice Reaction Task (reaction time), the Stroop Test (cognitive flexibility and inhibitory control), and the Trail Making Test (processing speed, cognitive flexibility, and visuoperceptual tracking) twice per day for five consecutive days (accounting for intraday and interday reliability). There are no universally accepted guidelines or recommendations on the number of habituation sessions required to minimize learning effects. However, White et al. (2019) suggested that more than two occasions were necessary. Thus, an exploratory approach was taken.

To improve the validity of reliability analyses in neurocognitive research and psychological measurements assessing executive and lower order functions, we supplemented common relative reliability statistics with advanced measurement error analyses. Specifically, in addition to the ICC, standard error of measurement (SEM), and minimal detectable change (MDC), an agreement analysis based on Bland and Altmann (1986), Hopkins (2000), and Atkinson and Nevill (1998) was conducted to evaluate random scattering (secondary variance) using the limits of agreement as a measure of precision (Barnhart et al. 2007). Furthermore, learning effects were evaluated using the sampled *t* test, as proposed by Atkinson and Nevill (1998) and Warneke et al. (2025), assuming that no systematic performance improvements should occur from test to retest if no learning und habituation effects were present (which is critical for internal data validity).

### 2.1 Participants

A sample size estimation for agreement analyses aimed at assessing reliability is difficult for two reasons: (i) G‐Power does not offer this option (correlations can be selected, but this is not valid for agreement analyses), and (ii) assuming high/maximal agreement would result in a very small sample size, potentially invalidating conclusive statistical procedures. Additionally, previous reliability and agreement studies did not perform post hoc sample size estimations (Dragutinovic et al. 2024; Feuerbacher et al. 2022) as these are generally seen as misleading (Dziak et al. 2020). To obtain a convenient and purposeful sample, we recruited 68 participants, of which 65 healthy participants successfully completed the study (age: 29.65 ± 20.67 years; height: 160.6 ± 15.5 cm; mass: 57.9 ± 20.1 kg). Assuming that children have different learning rates compared to adults, and adults compared to seniors, the sample was divided into three subgroups. Based on current age group classifications (Geifman et al. 2013), Group 1 consisted of regular schoolchildren without any diagnosed neurodevelopmental disorders (13 female [f] and 9 male [m]; age: 9.23 ± 1.34 years; minimum 7 years, maximum 11 years; height: 142.8 ± 11.9 cm; mass: 35.9 ± 9.6 kg). Group 2 included 20 young adult university students (16f, 4m; age: 21.9 ± 3.51 years, from 18 to 26 years; height: 169.0 ± 7.2 cm; mass: 64.4 ± 10.5 kg). Deviating from current age guidelines (see Section 5), Group 3 included 23 older adults (13f, 10m; age: 55.91 ± 6.13 years, from 47 to 64 years; height: 170.4 ± 6.4 cm, 74.2 ± 14.5 kg).

Participants were eligible for inclusion if they were healthy and stated the absence of neurodegenerative and neuropsychiatric diseases. There were no restrictions regarding age, sex, or physical performance level. All participants were blinded to the study's objectives and results. Written informed consent for participation was obtained from all participants or, in the case of minors, from their parents. The study protocol was confirmed by the local ethical review board (No. GZ.39/163/63).

### 2.2 Testing

To avoid systematic learning effects caused by a fixed testing order, the protocol was performed in randomized sequence via Excel randomization function. Allocation was concealed until the last possible moment. Results of the previous tests were communicated to participants as early as finishing the last testing session to avoid competitive effects.

#### 2.2.1 Ruler Drop Test

For the Ruler Drop Test, participants attempted to catch a falling ruler as quickly as possible (del Rossi et al. 2014). They sat alongside a table, resting the elbow at a 90° angle on the surface, with an open hand positioned on the edge of the table. The researcher held the ruler in a vertical position, ensuring that the zero‐mark aligned with the top of the participant's thumb and forefinger before letting it drop. The participant then tried to catch the ruler as quickly as possible (Ferreira et al. 2021). The protocol was first explained by the evaluator. Participants were instructed to focus on their hand and were informed that the ruler could drop at any time after hearing the word “ready.” Participants then completed three practice trials to familiarize with the testing protocol. The official testing protocol then commenced, and each participant performed the test twice with their dominant hand. The dominant hand was identified as the one typically used to hold chopsticks or a knife (Goto et al. 2024). To ensure a consistent time interval for all participants during both test and retest, the evaluator used a metronome set to 60 beats per minute. A sequence was established whereby the ruler was dropped after 3 s (first trial) and 4 s (second trial) following the evaluator's prompt of “ready.” The sound of the metronome was audible only to the evaluator, who used earbuds. During each trial, the distance between the thumb and index finger at the moment of catching was recorded. A 50‐cm ruler was used, and if the participant dropped it, a score of 51 cm was recorded (Ferreira et al. 2021, 2024).

#### 2.2.2 Trail Making Test

The Trail Making Test was developed as an improvement to the Army Individual Test Battery (Soukup et al. 1998). This paper‐and‐pencil assessment requires participants to connect numbered dots from 1 to 25 (i.e., 1–2–3–4), with the dots randomly scattered. If participants make seven or more errors, they are deemed unable to complete the test. Performance is evaluated by measuring the time taken (in seconds) to finish each condition, as well as the total number of errors made. In this study, just the Trail Making Test A was administered, as it is easier for children to understand and perform, only involving connecting numbers in the correct sequence. This task is less complex for children compared to the Trail Making Test B, which involves alternating between numbers and letters. By using only this version of the test, we aimed to ensure that children could successfully complete the task without being overwhelmed by the additional complexity.

#### 2.2.3 Stroop Test

In the traditional Stroop task (Stroop 1935), color names are presented in ink that do not correspond to the word's meaning, such as the word “green” displayed in red ink. This task has contributed substantially to our understanding of adult cognitive processes, attentional control mechanisms, and the neural bases of cognition. However, its use with children has been limited by the requirement that participants must have proficient reading skills (Prevor and Diamond 2005). The protocol was performed as described in previous studies (Wilke et al. 2020; Wilke and Vogel 2020). The test was slightly modified to create 10 different versions, ensuring that the exact same test was not administered 10 times in a row and that the word order differed. In detail, each participant entered the experimental room a few minutes before beginning the tasks, allowing their eyes to adjust to the ambient light (Stroop 1935). Color words, such as “green,” were displayed in incongruent ink colors, like “red.” Participants were instructed to quickly read through 50 words, while ignoring the color of the ink. To ensure valid test results for all participants, especially the children, we focused on the word‐reading task and omitted the color‐naming task, as naming ink colors often presents greater difficulty than reading a color patch. This decision was further supported by a pretest conducted with 12 children (ages 6–10) by the research team. In this study, both the time taken to complete the word‐reading task and the number of errors were recorded.

#### 2.2.4 Choice Reaction Task

**FIGURE 1:**
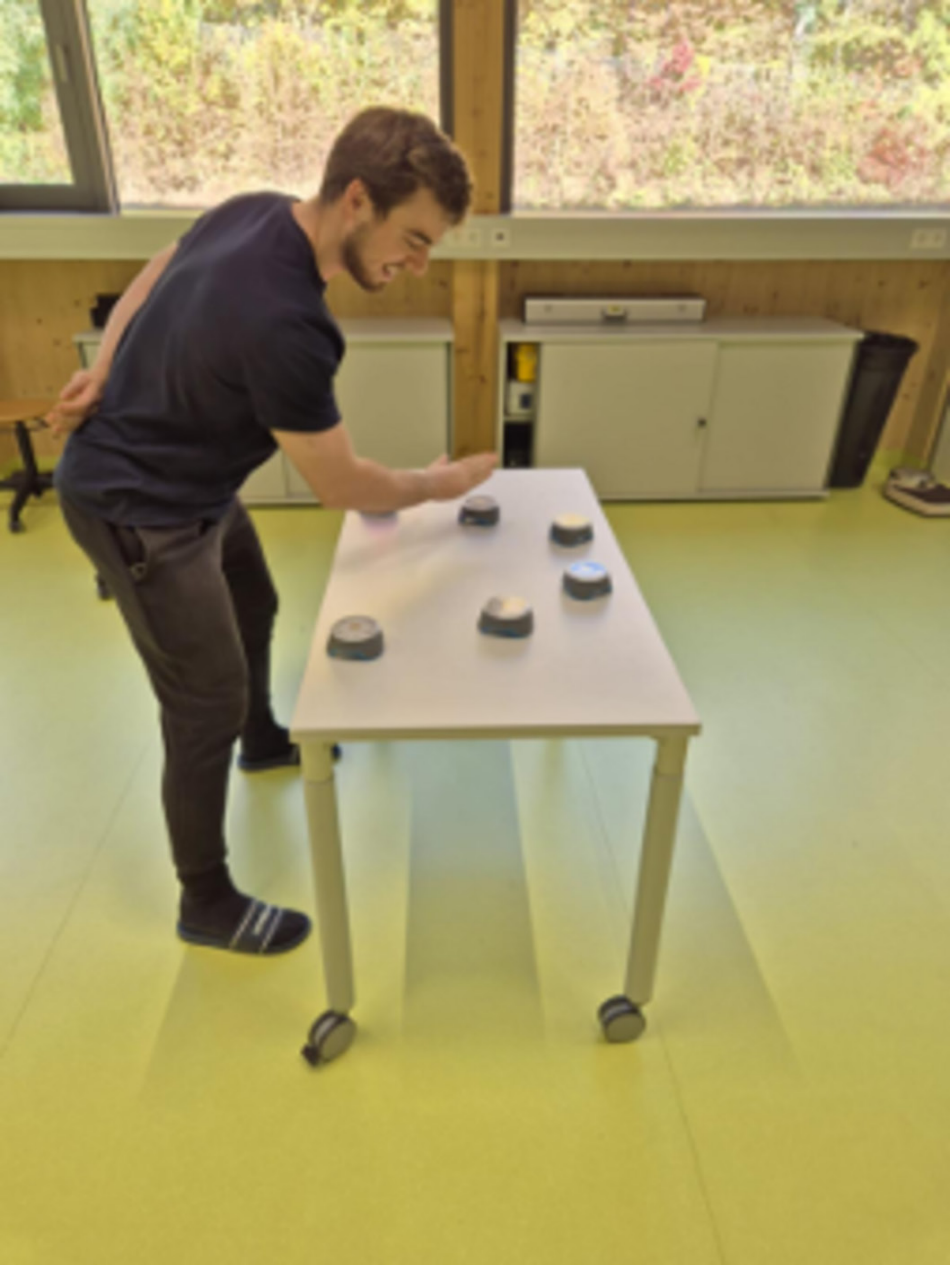
Showing exemplary the testing protocol for the Blazepod testing.

Response times were recorded and analyzed using the BlazePod system (BlazePod Inc., Miami, FL, United States). The setup comprised pods with light‐emitting diodes (LEDs) that illuminate randomly and deactivate upon being tapped (Figure 1). Participants first completed an introductory session to become familiar with the BlazePod system, followed by three to five practice trials (Chander et al. 2023). Six BlazePods were arranged in an inverted “U” shape on a table, with participants standing in front of the setup and beginning each trial at the software's prompt. Each participant performed two trials, using only their dominant hand. The color function was set to random, and each trial lasted 30 s. Both the number of hits within the 30‐s interval and the reaction times, as measured by the device, were included in the statistical analysis.

### 2.3 Hypothesis Testing

Given its frequent application in practice and the extensive prior research, one might expect testing to be reliable between scores from each day (separated hypotheses for interday reliability: Day 1 vs. Day 2, Day 2 vs. Day 3, etc.), while also assessing intraday reliability and repeatability on each testing occasion (Schmidt et al. 2018). However, a lack of reliability, such as that caused by systematic learning effects, reduces internal data validity as the test would monitor the ability to learn the tested function instead of the actually intended ability that was to be tested (Hopkins 2000; Warneke et al. 2025). This assumption applies to each individual comparison. Therefore, we aimed to test separated hypotheses: interday test–retest reliability between Days 1 and 2 as one hypothesis and between Days 2 and 3 as another. For each day‐to day comparison, it is essential that no significant differences are observed. Therefore, the alternative hypotheses to be evaluated via statistical tests were that

1. From day to day, there will be no significant systematic errors.
2. From day to day, the secondary variance/random error will remain stable.

This applies to all performed neurocognitive tests, including the Stroop Test, the Trail Making Test, and the Choice Reaction Task.

### 2.4 Statistics

Statistical analysis were conducted using JASP (Version 0.18.3 [Intel], 2024). The assumption of normality was verified using the Shapiro–Wilk test. Results are presented as both overall and age‐specific means (M) and standard deviations (SD), along with further analytical insights. Reliability was assessed for interday and intraday comparisons. For interday reliability, the average of the two valid trials from each test was calculated, comparing the mean of test Values 1 and 2 on Day 1 with the mean of test Values 3 and 4 on Day 2. Intraday reliability was evaluated based on the two valid trials conducted within a single day (comparison of Test 1 with Test 2 on Day 1).

Following guidelines from previous literature (Koo and Li 2016), two‐way ICCs for agreement, along with 95% confidence intervals, were calculated as follows:

$$\mathrm{ICC}={\mathrm{MS}}_{R}-{\mathrm{MS}}_{E}/\left({\mathrm{MS}}_{R}+\left({\mathrm{MS}}_{C}-{\mathrm{MS}}_{E}\right)/n\right),$$

where *ICC* is the intraclass correlation coefficient, ${\mathrm{MS}}_{C}$ represents the mean square for columns, ${\mathrm{MS}}_{E}$ denotes the mean square for error, ${\mathrm{MS}}_{R}$ is the mean square for rows, and *n* indicates the number of subjects.

Based on these calculations, the standard error of measurement (SEM) and the minimal detectable change were calculated, using (Tighe et al. 2010)

$$\mathrm{SEM}=\mathrm{SD}*\sqrt{1-\mathrm{ICC}},$$

where *SEM* is the standard error of measurement, *SD* represents the standard deviation of the mean difference between Trials 1 and 2, and *ICC* is the intraclass correlation coefficient.

The MDC was the calculated using (Powden et al. 2015; Seamon et al. 2022)

$$\mathrm{MDC}=\mathrm{SEM}*1.96*\sqrt{2},$$

where *MDC* is the minimal detectable change and *SEM* is the standard error of measurement.

Individual hypothesis testing for significant learning effects justifies the use of paired‐sampled *t* tests for interday and intraday test–retest comparisons (e.g., between Tests 1 and 2 or between Tests 3 and 4 for intraday reliability assessment). To examine the potential effects of age groups, a two‐way ANOVA with repeated measures was conducted to assess significant Time and Group * Time interactions. The Scheffé post hoc test was applied to confirm the results and adjust for α error. The magnitude of mean differences is further visualized in Bland–Altman plots, as recommended for reliability assessments (Grgic et al. 2020). These plots display the variability around a systematic shift (Atkinson and Nevill 1998; Bland and Altmann 1986; Hopkins 2000). To enhance the analysis of the limits of agreement using the mean difference as a reference, random error was also quantified through the mean absolute error (MAE) (Willmott and Matsuura 2005) and mean absolute percentage error (MAPE) (Kim and Kim 2016), calculated as follows:

$$\mathrm{MAE}=\frac{1}{n}*\sum_{i=1}^{n}\left|x_{i}-y_{i}\right|,$$

where *n* is the number of data points, *i* is the index for each paired data point, *x*_*i*_ represents the *i*th data point in variable *x* (e.g., Stroop Test Trial 1), and *y*_*i*_ represents the *i*th data point in variable *y* (e.g., Stroop Test Trial 2).

Similarly, MAPE was calculated as

$$\text{MAPE}=\frac{1}{n}*\sum_{i=1}^{n}\left|\frac{x_{i}-y_{i}}{x_{i}}\right|*100,$$

where *n* is the number of data points, *i* is the index for each paired data point, *x*_*i*_ represents the *i*th data point in variable *x* (e.g., Stroop Test Trial 1), and *y*_*i*_ represents the *i*th data point in variable *y* (e.g., Stroop Test Trial 2).

To provide a qualitative assessment of measurement variability and to track changes across testing sessions, Bland–Altman plots were included in the analysis. These plots display mean bias and limits of agreement (LoA), allowing a direct comparison of measurement errors between testing sessions. The ICCs were interpreted according to (Koo and Li 2016): Values below 0.50 indicate poor reliability; values from 0.50 to 0.75 indicate moderate reliability; values from 0.75 to 0.90 indicate good reliability; and values above 0.90 indicate excellent reliability. ANOVA results were interpreted as small if *η*_*p*_^*2*^ < 0.06, moderate if *η*_*p*_^*2*^ = 0.06 to < 0.14 and large if *η*_*p*_^*2*^ > 0.14. For post hoc comparisons, effect sizes were interpreted as trivial for *d* < 0.2, small for *d* = 0.2 to < 0.5, moderate for *d* = 0.5 to < 0.8, and large for *d* > 0.8 (Cohen 1988). The level of significance was set to α = 0.05. A Bonferroni–Holm correction was applied to adjust the *p* value according to the number of tests performed, ensuring accurate control over Type I error rates for each specific research question.

## 3 Results

Normal distribution of the results was confirmed using the Shapiro–Wilk Test, with *p* > 0.05 across all variables. To control for Type I error, the α‐error level for interday and intraday reliability was adjusted by dividing the total number of statistical tests (*n = 25*), resulting in a corrected significance threshold of 0.002. Interday and intraday reliability was treated as separated hypotheses for this adjustment.

### 3.1 Intraday Reliability

Intraday ICCs ranged from 0.39 to 0.97, indicating reliability levels from low to excellent, depending on the specific test (Koo and Li 2016). The lowest ICC values were observed for the Ruler Drop Test (0.39–0.75), whereas higher reliability was noted in the Stroop Test, Trail Making Test, and Choice Reaction Task, each with ICCs of ≥ 0.90. For the Ruler Drop Test, no significant systematic errors were identified between trials within the same day, but random errors ranged from 39.8% to 110.8%. In contrast, significant mean differences between intraday trials were observed for the remaining tests on the first testing days (*p* < 0.001 to *p* = 0.001). Depending on the performed tests, these mean differences diminished and became nonsignificant after 2–4 days (see Table 1).

**TABLE 1:** Intraday reliability metrics for each testing occasion. “Value 1” refers to the first test conducted within the day, whereas “Value 2” represents the second (retest) conducted in the same day. Statistical parameters—including the ICC, SEM, MDC, systematic error, MAE, and MAPE—were calculated between these two tests. The first test was used as the reference for MAPE calculations.

Intraday reliability								
Test	Mean ± SD (Value 1)	Mean ± SD (Value 2)	ICC (95% CI)	SEM	MDC	Systematic bias (level of sig.)	MAE	MAPE
Rulerdrop 1	20.9 ± 9.90	18.6 ± 8.90	0.75 (0.59–0.84)	4.24	7.01	2.33 (0.03)	6.32	39.84
Rulerdrop 2	16.2 ± 8.15	14.7 ± 5.71	0.71 (0.54–0.82)	4.32	11.99	1.50 (0.07)	5.19	49.95
Rulerdrop 3	13.0 ± 7.03	12.4 ± 7.39	0.64 (0.42–0.77)	4.47	12.40	0.54 (0.56)	5.15	56.59
Rulerdrop 4	12.9 ± 7.68	11.1 ± 6.19	0.63 (0.41–0.77)	4.42	12.25	1.81 (0.05)	5.73	110.78
Rulerdrop 5	11.2 ± 6.02	10.6 ± 6.72	0.39 (0.03–0.62)	6.16	17.06	0.55 (0.57)	5.8	110.81
Trail Making Test 1	32.4 ± 14.53	23.4 ± 10.53	0.92 (0.87–0.95)	1.98	5.48	8.83 (< 0.001)	9.19	40.5
Trail Making Test 2	25.7 ± 10.61	21.6 ± 9.63	0.93 (0.89–0.96)	1.35	3.73	4.10 (< 0.001)	5.10	25.68
Trail Making Test 3	21.9 ± 10.37	18.6 ± 7.94	0.91 (0.86–0.94)	1.59	4.41	3.29 (< 0.001)	4.38	23.38
Trail Making Test 4	17.1 ± 8.31	15.1 ± 7.06	0.94 (0.90–0.96)	0.91	2.52	2.04 (< 0.001)	2.93	18.82
Trail Making Test 5	16.8 ± 6.93	14.7 ± 6.41	0.96 (0.93–0.97)	0.55	1.53	2.09 (< 0.001)	2.50	18.79
Stroop Test 1	24.4 ± 9.31	22.3 ± 7.96	0.97 (0.96–0.98)	0.49	1.35	2.12 (< 0.001)	2.46	10.66
Stroop Test 2	22.6 ± 8.67	21.5 ± 7.65	0.98 (0.96–0.98)	0.36	1.00	1.09 (0.001)	1.56	6.64
Stroop Test 3	20.6 ± 6.55	20.3 ± 6.57	0.99 (0.98–0.99)	0.15	0.41	0.30 (0.11)	1.22	6.23
Stroop Test 4	19.4 ± 5.84	19.3 ± 5.73	0.97 (0.95–0.98)	0.36	0.99	0.14 (0.58)	1.20	6.25
Stroop Test 5	19.3 ± 5.61	18.42 ± 5.79	0.98 (0.97–0.99)	0.23	0.63	−0.08 (0.69)	1.20	6.22
Choice Reaction Task1	43.0 ± 7.17	45.3 ± 6.97	0.97 (0.95–0.98)	0.54	1.51	−2.35 (< 0.001)	2.72	6.10
Choice Reaction Task 2	46.2 ± 6.69	47.6 ± 7–34	0.95 (0.92–0.97)	0.60	1.67	−1.42 (< 0.001)	2.50	5.54
Choice Reaction Task 3	48.7 ± 6.73	49.3 ± 7.13	0.97 (0.95–0.98)	0.42	1.18	−0.55 (0.07)	2.0	4.18
Choice Reaction Task 4	49.9 ± 6.77	50.4 ± 6.48	0.96 (0.93–0.97)	0.54	1.48	−0.54 (0.11)	2.05	4.20
Choice Reaction Task 5	50.3 ± 6.47	50.8 ± 6.61	0.96 (0.94–0.98)	2.38	6.58	−0.46 (0.13)	1.85	3.78
Reaction Time 1	586.1 ± 132.14	548.2 ± 113.72	0.96 (0.93–0.97)	9.82	27.23	37.86 (< 0.001)	44.35	7.93
Reaction Time 2	532.9 ± 98.25	516.2 ± 110.12	0.92 (0.88–0.95)	15.71	43.56	16.72 (0.02)	38.75	7.11
Reaction Time 3	497.9 ± 84.87	494.8 ± 96.60	0.96 (0.93–0.97)	7.28	20.19	3.03 (0.51)	26.32	5.12
Reaction Time 4	484.8 ± 82.84	473.9 ± 79.81	0.95 (0.91–0.97)	8.27	22.93	10.91 (0.02)	27.95	5.71
Reaction Time 5	476.9 ± 81.20	472.6 ± 80.89	0.96 (0.94–0.98)	1.26	3.48	4.25 (0.28)	22.65	4.59

Abbreviations: 1, Day 1; 2, Day 2; 3, Day 3; 4, Day 4; 5, Day 5; ICC, intraclass correlation coefficient; MAE, mean absolute error, MAPE, mean absolute percentage error; MDC, minimal detectable change, mean, mean value at the respective day; SD, standard deviation; SEM, standard error of measurement; Value 1, value of the first test at the day; Value 2, value of the retest on the same day.

A reduction in random errors was observed across tests over the five testing days, indicating decreased secondary variance. For example, random errors in the Choice Reaction Task decreased from over 6% on Day 1 to 3.8% by Day 5. Similarly, the Trail Making Test demonstrated a meaningful reduction in MAPE, from 40.5% on Day 1 to 18.8% by the final testing day. These tendencies can be exemplarily described for the Stroop test. The systematic bias (mean difference) was significant for the first two testing occasions (*p* < 0.001 on Day 1 and *p* = 0.001 on Day 2) but stopped being significant by Day 3 (*p* = 0.11 on Day 3, *p* = 0.58 on Day 4, and *p* = 0.69 on Day 5). The MAPE decreased from Day 1 with > 10%–6.22% on Day 5. For the Trail Making Test, in contrast, there were significant intraday improvements over all five testing days observed from test to retest (*p* < 0.001), whereas the random error started at > 25% and decreased to about 19% on the fifth testing day.

### 3.2 Overall Interday Reliability

**FIGURE 2:**
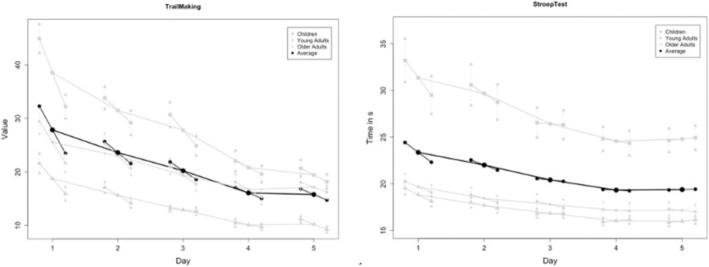
Learning curves for the TMT and Stroop tests from Days 1 to 5, shown for the overall group (black) and the different age groups (gray) accounting for the intraday testing variance (vertical point order) and the interday course, connected horizontally. Vertical lines show the standard error of measurement.

Similar results were found for interday reliability. With *ICC* ranging between 0.48 and 0.81, the Ruler Drop Test had the worst interday reliability; the other tests had *ICC*s between 0.77 and 0.99. Significant systematic changes from test to test were observed across all tests from day to day (see Figure 2), generally diminishing after the second to fourth testing day. Specifically, these systematic effects subsided after the second day for the Ruler Drop Test and after the third day for the Trail Making Test and Choice Reaction Task. The Stroop Test and the Choice Reaction Task showed no significant mean differences after the fourth to fifth day. All tests demonstrated significant changes from Testing Days 1 to 5 (*p* < 0.001).

Similar to the results obtained for intraday reliability, random error was highest in the Ruler Drop Test (≥ 48%) and lowest in the Choice Reaction Task (3.25%) in day‐to‐day comparisons. Nevertheless, the random error decreased from day to day in most cases (with few exceptions). For the Stroop Test, the MAPE consistently diminished from > 11% between Days 1 and 2 to under 5% between Days 4 and 5. In the Choice Reaction Task, MAPE diminished from more than 6% to about 3% in between the final testing days. This tendency was less clear for the Trail Making Test and the Ruler Drop Test. Although there was a MAPE of 42.80% between the first testing days, this value increased to 44.50% between the second and third days and to 48.57% between the fourth and fifth testing days in the Ruler Drop Test. In the Trail Making test, over the first two testing occasions, there was a reduction from 26.60% to 23.31%, but for the third to fourth testing session, the MAPE increased to 31.01%.

**FIGURE 3:**
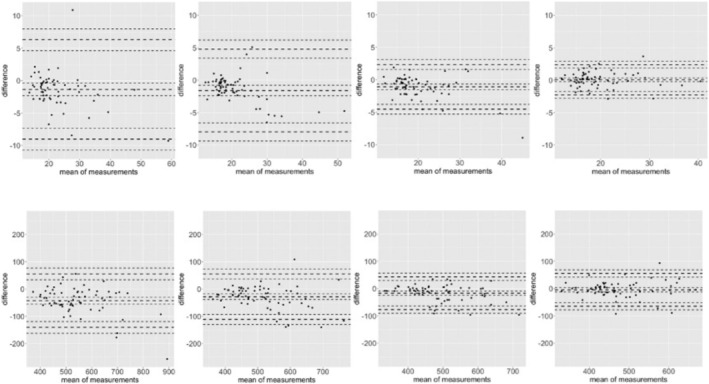
Bland–Altman Analysis accounting for the mean systematic bias (mean difference illustrated via the midline) as well as the limits of agreements (upper and lower border including their respective 95% CIs of the test–retest differences in dependency of the mean of the measurements; please review Warneke et al. 2025 and Lohmann et al. 2024 for more details) for the choice reaction task (CRT) and the reaction time of the CRT. The graphic illustrates how the mean difference as well as the random error decreases just due to getting familiar with the testing conditions, as no intervention was applied.

Comparing the mean values from Day 1 (average of Tests 1 and 2 on Day 1) to Day 5, the Ruler Drop Test exhibited the highest MAPE at 121.56%, indicating substantial random error. The Trail Making Test, Stroop Test, and Choice Reaction Task also showed considerable random errors with MAPE values of 81.86%, 20.43%, and 12.87%, respectively. In accordance with Warneke et al. (2025), these findings underscore the presence of meaningful random errors across all tests throughout the testing period (see Table 2 and Figure 3), even though most of the *ICC*s indicated sufficient reliability.

**TABLE 2:** Interday reliability metrics calculated using the mean of the test–retest values within each testing occasion. Statistical parameters—including ICC, SEM, MDC, systematic error, MAE, and MAPE—were calculated between testing days, with the earlier testing day used as the reference for MAPE calculations.

Test	Mean ± SD (Value 1)	Mean ± SD (Value 2)	ICC (95% CI)	SEM	MDC	Systematic bias (level of sig.)	MAE	MAPE
Rulerdrop (in cm) 1_2	19.8 ± 8.40	15.4 ± 6.19	0.75 (0.61–0.85)	3.28	9.11	4.41 (< 0.001)	5.69	42.80
Rulerdrop (in cm) 2_3	15.4 ± 6.19	12.7 ± 6.17	0.79 (0.67–0.87)	2.34	6.50	2.70 (< 0.001)	4.47	44.50
Rulerdrop (in cm) 3_4	12.7 ± 6.17	12.0 ± 5.96	0.81 (0.70–0.88)	2.09	5.81	0.72 (0.23)	3.55	38.34
Rulerdrop (in cm) 4_5	12.0 ± 5.96	10.9 ± 5.02	0.69 (0.50–0.80)	3.00	8.31	1.06 (0.12)	4.20	48.57
Rulerdrop (in cm) 1_5	19.8 ± 8.40	10.9 ± 5.02	0.48 (0.17–0.68)	5.28	16.15	8.89 (< 0.001)	9.57	121.56
Trail Making Test in s 1_2	27.9 ± 12.20	23.6 ± 9.81	0.90 (0.85–0.94)	2.07	5.74	4.24 (< 0.001)	5.75	26.60
Trail Making Test in s 2_3	23.6 ± 9.81	20.2 ± 8.85	0.94 (0.90–0.96)	1.11	3.09	3.39 (< 0.001)	4.37	23.31
Trail Making Test in s 3_4	20.2 ± 8.85	16.1 ± 7.48	0.93 (0.89–0.96)	1.11	3.07	4.15 (< 0.001)	4.55	31.01
Trail Making Test in s 4_5	16.1 ± 7.48	15.8 ± 6.53	0.96 (0.93–0.97)	1.31	3.62	0.29 (0.42)	1.98	12.25
Trail Making Test in s 1_5	27.9 ± 12.20	15.8 ± 6.53	0.77 (0.84–0.86)	1.37	3.84	12.08 (< 0.001)	12.19	81.86
Stroop Test in s 1_2	23.4 ± 8.54	22.0 ± 8.07	0.94 (0.91–0.96)	0.96	2.66	1.36 (0.007)	2.56	11.16
Stroop Test in s 2_3	22.0 ± 8.07	20.4 ± 6.52	0.95 (0.92–0.97)	0.73	2.02	1.60 (< 0.001)	2.04	9.17
Stroop Test in s 3_4	20.4 ± 6.52	19.3 ± 5.69	0.98 (0.97–0.99)	0.23	0.63	1.09 (< 0.001)	1.41	7.06
Stroop Test in s 4_5	19.3 ± 5.69	19.4 ± 5.64	0.99 (0.98–0.99)	0.12	0.33	−0.058 (0.70)	0.89	4.66
Stroop Test in s 1_5	23.4 ± 8.54	19.4 ± 5.64	0.92 (0.87–0.95)	1.35	3.73	3.99 (<0.001)	4.15	20.43
Choice Reaction Task 1_2	44.1 ± 6.95	46.8 ± 6.86	0.96 (0.94–0.98)	0.52	1.43	−2.73 (< 0.001)	3.1	6.63
Choice Reaction Task 2_3	46.8 ± 6.86	49.0 ± 6.83	0.96 (0.94–0.98)	0.44	1.23	−2.15 (<0.001)	2.56	5.34
Choice Reaction Task 3_4	49.0 ± 6.83	50.2 ± 6.49	0.97 (0.96–0.98)	0.37	1.03	−1.13 (< 0.001)	1.76	3.62
Choice Reaction Task 4_5	50.2 ± 6.49	50.6 ± 6.42	0.97 (0.95–0.98)	0.40	1.11	−0.42 (0.14)	1.6	3.25
Choice Reaction Task 1_5	44.1 ± 6.95	50.6 ± 6.42	0.89 (0.83–0.93)	1.39	3.86	−6.44 (< 0.001)	6.5	12.87
Reaction Time 1_2	567.2 ± 120.8	524.5 ± 100.6	0.95 (0.92–0.97)	11.16	30.94	42.65 (< 0.001)	48.17	9.16
Reaction Time 2_3	524.5 ± 100.6	496.4 ± 89.09	0.95 (0.92–0.97)	9.51	26.36	28.17 (< 0.001)	35.86	7.04
Reaction Time 3_4	496.4 ± 89.09	479.4 ± 79.21	0.97 (0.95–0.98)	5.32	14.73	16.99 (< 0.001)	24.06	4.89
Reaction Time 4_5	479.4 ± 79.21	474.8 ± 79.51	0.96 (0.94–0.98)	6.12	16.97	4.61 (0.23)	21.47	4.47
Reaction Time 1_5	567.16 ± 120.8	474.75 ± 79.51	0.82 (0.72–0.89)	33.72	93.46	92.41 (< 0.001)	93.67	19.78

Abbreviations: 1, Day 1; 2, Day 2; 3, Day 3; 4, Day 4; 5, Day 5; ICC, intraclass correlation coefficient; MAE, mean absolute error, MAPE, mean absolute percentage error; MDC, minimal detectable change, mean, mean value at the respective day; SD, standard deviation; SEM, standard error of measurement; Value 1, value of the first test at the day; Value 2, value of the retest on the same day.

Bland–Altman analyses provide further viable insights, illustrating results for the mean differences (systematic bias) and showing a considerable decrease in the limits of agreement (*LoA*) range from test to test for the Choice Reaction Task as well as the Reaction Time in the Choice Reaction Task (Figure 2).

### 3.3 Age‐Specific Effects

The two‐way *ANOVA*, with Scheffé post hoc tests, revealed significant baseline differences across age groups, as shown in Figure 2. Children consistently demonstrated the lowest baseline values across tests. In the Ruler Drop Test, children scored significantly lower than both adult groups (*p* < 0.001, *d =* 2.24), whereas older adults showed reduced performance compared to younger adults (*p =* 0.04, *d* = 0.78). Similar age‐specific baseline differences were observed for the Trail Making Test, indicating the worst performance in children (*p* < 0.001, *d* = 2.16 compared to adults), whereas the difference between young adults and older adults slightly failed to reach significance (*p* = 0.06).

For the Choice Reaction Task performance, children (*p* < 0.001, *d* = 2.75) and older adults (*p* = 0.007, *d* = 1.0) performed significantly worse compared to the young adults. For Choice Reaction Task *reaction time*, an effect was observed between children and young adults with *p* < 0.001, *d* = 2.4, and between children and older adults with *p <* 0.001, *d* = 1.93. In contrast, the Stroop test indicated no differences between young and older adults (*p =* 0.91), but there were large differences between children and both adult groups (*p* < 0.001, *d* = 1.93).

The one‐way ANOVA performed between the first and last day indicated significant performance increase between the children and adults (young and older) for the Ruler drop test, the Trail Making test, the Stroop test, and the reaction time of the Choice Reaction Task (*p <* 0.001–0.004), indicating significant learning effects over all groups. In contrast, no differences between changes from the first to last day were observed between the groups for the Choice Reaction Task performance (*p* = 0.33–0.86). If available, all tests showed significantly higher performance improvements in children. Only in the Ruler Drop Test did posttest group comparisons fail to show significant differences between children and the adult groups. In the remaining tests, the performance level of children remained below that of the adults.

None of the tests showed significant age‐specific ICC differences (95% confidence intervals overlapped), with similar random errors across the age groups. For instance, for the Stroop Test, the interday reliability ranged from 0.88 to 0.99 for all age groups; a maximum MAPE was reported at approximately 27% in the children, whereas young and older adults had around 17% and 18%, respectively (see Table S1–S3). This tendency was observed for all testing procedures, showing similar ICCs, whereas the random scattering was higher in children compared to the other groups. This phenomenon was not confirmed for intraday reliability: Although ICCs were similar (and without significant differences) between the age groups, no clear tendency could be observed for the MAPE. For instance, whereas in the Trail Making Test, children showed the highest MAPEs with 42.09%, young adults also reached a MAPE of 41.82% on the first testing day. For more detailed age‐specific reliability statistics, the reader is referred to the Supplemental Material (Tables S1–S3).

## 4 Discussion

Our goal was to investigate the reliability and magnitude of errors in frequently applied neurocognitive tests used to assess cognitive flexibility and inhibitory control (Stroop Test), reaction time (Ruler Drop Test and Choice Reaction Task), and orientation and visual scanning (Trail Making Test). In accordance with previous studies, most tests exhibited sufficient relative reliability (e.g., ICC). However, our findings revealed significant systematic and random measurement errors, in some instances reaching approximately 120%. These errors can largely be attributed to group‐specific learning/habituation effects within the first four testing days, highlighting previous calls for more extensive familiarization procedures when testing for neurocognitive capacity (White et al. 2019). Importantly, apart from reduction in the systematic day‐to‐day difference that could indicate meaningful learning and habituation effects, habituation to testing conditions was not confined solely to reducing systematic bias by time. The random error, as quantified by the MAE and MAPE and visualized via the Limits of Agreement from the Bland–Altman analysis, also decreased, and the 95% CIs narrowed in the graphical illustration (see Figure 3). Therefore, in this specific case and for these participants, four testing sessions were necessary to reduce the systematic bias to nonsignificant levels (which does not automatically mean it is practically irrelevant) and to meaningfully reduce random error. Poor reliability (e.g., due to missing familiarization) is a crucial parameter for internal data validity and could indicate learning and habituation effects. Therefore, and in accordance with previous suggestions (White et al. 2019), our study underscores the relevance of a sufficient number of habituation sessions, showing that a single session may be insufficient. Otherwise, it cannot be ruled out that the tests do not measure the intended construct (e.g., inhibitory control) but rather participants' familiarity with the tasks and their learning/habituation rates in adapting to the testing protocol.

### 4.1 Lack of Control for Habituation Effects and Random Scattering in Neurocognitive Research

Given the growing interest in studies examining baseline neurocognitive function and the effects of various interventions, it is notable that systematic reviews with meta‐analysis on reliability of the explored cognitive tests are lacking. Studies utilizing these tests often do not adequately account for the various facets of measurement error. For example, Takahashi and Grove (2020) reported *ICC*s ranging from 0.75 to 0.90 for the Stroop neutral and incongruent test, frequently employed to evaluate visual search speed, working memory (Periáñez et al. 2021), and inhibitory control (Laskov et al. 2023). These *ICC*s are below what we found in our data collection. To mitigate learning effects, Takahashi and Grove (2020) counterbalanced the session order. However, this approach reflects a limited understanding of learning and habituation effects, which extend beyond just session order and may, in fact, negatively influence results due to mental fatigue. Although a randomized order can reduce intertest influence, it does not counteract learning or day‐to‐day habituation effects. Because there was no assessment of systematic and random measurement errors, potential habituation effects in this population were overlooked. Similarly, Giesche et al. (2020) conducted several neurocognitive tests and acknowledged learning effects through habituation trials in general, but the authors did not quantify reliability. Strauss et al. (2005) investigated interday *ICC*s ranging from 0.63 to 0.94, which aligned with our findings on relative reliability. However, systematic learning effects or random scattering (sources of secondary variance) were not addressed.

Relative reliability indices, although informative, are insufficient without corresponding systematic and random error quantifications. In a recent review, Warneke et al. (2025) showed that *ICC*s *>* 0.9 (Koo and Li 2016) can be accompanied by significant interday mean differences and show random error of > 20%. Another caveat in the literature is that studies often rely solely on previously reported relative reliability coefficients to justify the validity of their own measurement protocols, without providing their own reliability statistics. (Engeroff et al. 2019; Wilke et al. 2020; Wilke and Vogel 2020; Wöstmann et al. 2013; who referred to, e.g., Galpin et al. 2008). Therefore, the distinction between the reliability of a measurement protocol and test reliability is often overlooked. Devices and tests (e.g., Fitlights, Blazepods or MRI devices, the Stroop test, and the Trail Making Test) may reliably measure specific parameters; however, biological and subjective factors preclude generalizable reliability across different protocols and populations. In our study, this is evident from the varying learning rates observed among age groups and underlines the need to distinguish between protocol/device reliability and the actual testing reliability. Consequently, the *ICC* is inadequate for measurement errors and learning effects (Hopkins 2000; Atkinson and Nevill 1998), and no universal testing protocol reliability can be generalized from one trial to another. Similar issues arise in reliability determinations for the protocols involving the Trail Making Test, the Choice Reaction Task (De‐Oliveira et al. 2021; Wilke and Vogel 2020), or the Ruler Drop Test (Ángel Latorre‐Roman et al. 2018), with prior research also highlighting poor reliability of the Ruler Drop Test measures (Ferreira et al. 2024).

In accordance with previous literature (Zorowitz and Niv 2023), lack of reliability questions the validity of neurocognitive tests and suggests that extensive habituation training is required before performing such tests (White et al. 2019). Our results demonstrate a stabilization in our population after approximately four testing sessions with two tests, although this duration may vary across participant groups and testing conditions. This stabilization was reflected in the absence of any further systematic error beyond the fourth session, with progressively narrower the *LoA*s in Bland–Altman plots as participants gained testing experience. We aim to raise awareness among future researchers for the need to control for learning and habituation effects to yield valid and reliable study results. Although we found a lack of systematic mean difference between the fourth and fifth testing sessions, statistically nonsignificant does not mean clinically irrelevant (or vice versa). Furthermore, because we stopped the testing sessions after the fifth day, we cannot make any conclusions about the learning and habituation curve that could have followed (i.e., maybe reliability increases further until Day 7 or 8). In any case, researchers and clinicians must find the split between practical applicability and maximal stability and reliability in tests. With an increasing number of habituation sessions, the commitment of participants to participate in numerous tests before starting the actual testing protocol might decrease. Nevertheless, we want to raise awareness to habituation and learning effects and their relevance for data interpretation, which enhances the relevance to implement several habituation sessions, even if it requires greater time and effort to ensure accurate testing conditions. Although extensive habituation may reduce effect magnitudes, rigorous study designs under controlled testing conditions are essential.

### 4.2 Age Effects

The need to avoid generalizations of reliability across populations is underscored by the fact that different populations may exhibit varying learning rates, especially in unfamiliar cognitive tasks. Vaportzis et al. (2013) observed that older participants, while equally accurate, were slower than younger counterparts, highlighting both group and parameter specificity that affects the transferability of findings. Moreover, secondary variance among different populations has often been overlooked in reliability assessments. Bock and Girgenrath (2006) demonstrated significant variance differences in test performance between older and younger participants, suggesting age as a moderator of neurocognitive task heterogeneity and stability. Accordingly, our results show higher MAPE for the children and older adults compared to the younger adults (refer to Tables S1–S3). The analyses of variance suggested different learning rates, with children displaying faster learning but starting from a lower baseline. This raises questions about whether age or baseline performance levels primarily influence learning effects. The broader variability in baseline values and higher random errors among the young and older age groups indicate that although measurements were more stable among young adults, variability increased in both younger and older groups. It can be speculated that in the underaged, neurocognitive ability might undergo different development velocities, whereas in older adults, it is possible that neuro‐degeneration in some participants resulted in a broader performance range.

### 4.3 Measurement Errors Versus the Minimal Detectable Change (MDC)

When a pre–post change surpasses the calculated *MDC*, this adaptation is likely not attributable to testing errors. However, when reviewing the calculation formula (see Section 2.4), the *MDC* is strongly influenced by the *ICC*, as this value determines the *SEM*. Unfortunately, the *ICC* does not account for systematic measurement errors (Atkinson and Nevill 1998; Barnhart et al. 2007; Hopkins 2000). As shown in our results, high *ICC*s were partially accompanied by significant mean differences from repeated testing sessions, indicating substantial learning effects arising from repeated testing. Across several testing sessions, the systematic error from interday and intraday reliability surpassed the *MDC*, indicating that there was a significant training effect—however without an intervention performed. This raises the question whether multiple testing in neurocognitive tasks can be seen as an own intervention protocol, if participants are unfamiliar with the tasks. The *MDC* only provides valuable information if sufficient familiarization was performed in advance, which must be evaluated in each data collection session. When significant mean differences are observed between the test and retest, the *MDC* should be considered largely uninterpretable.

Of course, the urgency of such issues depends on the research questions and research design at hand. For example, when comparing the performance of two participants that perform a task once, we would need prior information on their familiarity with the task and related tasks, and the measurement error accuracy in relation to the expected difference between these participants. Another critical case might be where a single participant performs such tests with a single preassessment and postassessment. Here, we might also need information about prior experience with the test, and—as has been shown in this paper—we need to gauge the expected change in test scores that is likely based on repeated testing alone. Although a control group is commonly implemented into high‐quality research that should account for variability problems, it does not solve problems invalidity of testing in case of lack of reliability.

## 5 Limitations

This study aimed to investigate the reliability of selected neurocognitive tasks, providing deeper insights into population‐specific habituation effects and determining how many testing sessions are required to achieve a stable level of cognitive performance, thereby enabling valid conclusions. However, some limitations must be considered when interpreting the findings. First, one could argue that performing separated *t* tests may not have been the most statistical appropriate approach due to the potential accumulation of the Type I errors. However, our study design necessitated testing until no further systematic error was detected, which meant that the number of required tests was unknown a priori. Thus, each interday test–retest reliability must be treated as a stand‐alone hypothesis, which precludes the use of a single omnibus *ANOVA*. To counteract the potential α‐error accumulation, we adjusted the α‐error level by dividing 0.05/25 (testing occasions). Furthermore, a one‐way ANOVA for repeated measures confirmed significant mean transitions across the testing period (*η*_*p*_^2^ = 0.05–2, *p <* 0.001–0.03), thereby corroborating our more practical individual hypothesis testing approach using separate sample *t* tests.

A second limitation arises from the fact that we did not perform all possible versions of the implemented neurocognitive tests. According to Llinàs‐Reglà et al. (2017) and Scarpina and Tagini (2017), drawing valid conclusions about neurocognitive abilities ideally requires different versions of the Stroop test as well as both Trail Making Tests A and B. However, to ensure a consistent testing protocol across participants of the various age groups, we prioritized reducing the length of the measurement session. Extending the test duration risked introducing bias due to fatigue effects, particularly for the youngest participants. As the study did not seek to draw any conclusions about neurocognitive function classifications, performance ranking or evaluate intervention effects, this limitation does not compromise the validity of our results. Instead, our simplified protocol allowed us to effectively assess how habituation affected reliability. Nevertheless, future protocols should explore the implementation of comprehensive test versions (e.g., color reading, word reading, and Trail Making Tests A and B). Given that reliability coefficients are not transferable across populations, this broader testing approach will only provide additional value when applied to the specific population included in such studies.

Another potential limitation is the performance of 10 tests in five testing occasions. However, because no generally accepted guidelines exist, we used this as an exploratory approach with a still practically relevant number of testing sessions, surpassing the proposal of White et al. (2019). Whether additional improvements can be expected when performing further testing sessions remains speculative. The absence of systematic error after (mostly) four testings was perhaps a lucky coincidence, as no content‐based justification to perform five testing days was considered.

In this study, learning and habituation effects were equalized with statistically significant systematic mean differences, indicating a systematic performance improvement without related to an intervention. Nevertheless, we cannot distinguish between habituation effects or an increase in familiarity with the test and actual learning effects with building neuronal connections. Regardless, the results are still coherent with the study's goal, as we aimed to quantify different measurement error facets arising just from habituate to neurocognitive tests to raise awareness for the impact of reliability metrics on internal data validity.

Finally, although our sample size of 68 participants compares favorably to those of similar studies, previous research indicated that correlation‐based coefficients stabilize more reliably with larger sample sizes (Schönbrodt and Perugini 2013). This limitation may impact the validity of subgroup analyses, particularly because the maximum subgroup size in our study was 24 (older adult group). Although the overall sample size seems higher than in comparable studies, the subgroup analyses are biased due to limited sample sizes. Additionally, infrastructural limitations prevented the recruitment of participants classified as “older adults” based on the World Health Organization (WHO) standards (starting at 65 years of age) (Geifman et al. 2013; World Health Organization 2020). Future research should increase sample sizes within each subgroup to improve reliability estimates and include participants over 65 years old to enhance validity in age comparisons.

## 6 Conclusion

This study supports previous calls for more extensive habituation sessions, beyond the typical two, before evaluating neurocognitive performance capacity in individuals. Inconsistent testing conditions may fail to accurately reflect participants' true cognitive abilities. Our data indicate that systematic bias decreases from trial to trial, whereas the *LoA*s and random measurement error converge toward a “true” performance value. A suitable pretesting benchmark is reached when systematic bias becomes nonsignificant and the range of random error stabilizes. However, different populations exhibit varying learning rates and individual baseline values, making it impossible to generalize reliability indices across different populations or studies. Each data collection session must independently establish reliable measurements, as unreliable data inherently preclude valid performance assessments.

## Author Contributions

**Manuel Oraže:** data curation, investigation, writing – review and editing. **Gerit Plöschberger:** data curation, investigation. **Marco Herbsleb:** validation, writing – review and editing. **Jose Afonso:** conceptualization, methodology, validation, writing – review and editing. **Sebastian Wallot:** conceptualization, formal analysis, methodology, supervision, validation, writing – review and editing.

## Conflicts of Interest

The authors declare no conflicts of interest.

### Peer Review

The peer review history for this article is available at https://www.webofscience.com/api/gateway/wos/peer‐review/10.1111/ejn.70117.

## Supporting information


**Table S1** Reliability statistics for intra‐ and interday for all performed tests for the underaged group (children). Value one shows either the first test on the day and value to the second test on the day (intraday), or value 1 represents the mean within the first day and value to the mean within the second testing day (interday). The Intraclass Coefficient Correlation (ICC), Standard Error of Measurement (SEM), Minimal Detectable Change (MDC), systematic bias, Mean Absolute Error (MAE) and Mean Absolute Percentage Error (MAPE) were calculated between the two respective values, depending on the research question to be answered.
**Table S2** shows reliability statistics for intra‐ and interday for all performed tests for the young adults. Value one shows either the first test on the day and value to the second test on the day (intraday), or value 1 represents the mean within the first day and value to the mean within the second testing day (interday). The Intraclass Coefficient Correlation (ICC), Standard Error of Measurement (SEM), Minimal Detectable Change (MDC), systematic bias, Mean Absolute Error (MAE) and Mean Absolute Percentage Error (MAPE) were calculated between the two respective values, depending on the research question to be answered.
**Table S3** shows reliability statistics for intra‐ and interday for all performed tests for the older adults. Value one shows either the first test on the day and value to the second test on the day (intraday), or value 1 represents the mean within the first day and value to the mean within the second testing day (interday). The Intraclass Coefficient Correlation (ICC), Standard Error of Measurement (SEM), Minimal Detectable Change (MDC), systematic bias, Mean Absolute Error (MAE) and Mean Absolute Percentage Error (MAPE) were calculated between the two respective values, depending on the research question to be answered.
**FIGURE S1** Measurement procedure performed in this study

## Data Availability

Original data can be requested from the corresponding author due to reasonable request.
