# Supplementary material for: When Testing Becomes Learning—Underscoring the Relevance of Habituation to Improve Internal Validity of Common Neurocognitive Tests
Source: Eur J Neurosci. 2025 Apr 24;61(8):e70117. doi: 10.1111/ejn.70117 (PMC12022537; doi:10.1111/ejn.70117)
Supplement: Supplementary file 1 — Table S1 Reliability statistics for intra‐ and interday for all performed tests for the underaged group (children). Value one shows either the first test on the day and value to the second test on the day (intraday), or value 1 represents the mean within the first day and value to the mean within the second testing day (interday). The Intraclass Coefficient Correlation (ICC), Standard Error of Measurement (SEM), Minimal Detectable Change (MDC), systematic bias, Mean Absolute Error (MAE) and Mean Absolute Percentage Error (MAPE) were calculated between the two respective values, depending on the research question to be answered. Table S2 shows reliability statistics for intra‐ and interday for all performed tests for the young adults. Value one shows either the first test on the day and value to the second test on the day (intraday), or value 1 represents the mean within the first day and value to the mean within the second testing day (interday). The Intraclass Coefficient Correlation (ICC), Standard Error of Measurement (SEM), Minimal Detectable Change (MDC), systematic bias, Mean Absolute Error (MAE) and Mean Absolute Percentage Error (MAPE) were calculated between the two respective values, depending on the research question to be answered. Table S3 shows reliability statistics for intra‐ and interday for all performed tests for the older adults. Value one shows either the first test on the day and value to the second test on the day (intraday), or value 1 represents the mean within the first day and value to the mean within the second testing day (interday). The Intraclass Coefficient Correlation (ICC), Standard Error of Measurement (SEM), Minimal Detectable Change (MDC), systematic bias, Mean Absolute Error (MAE) and Mean Absolute Percentage Error (MAPE) were calculated between the two respective values, depending on the research question to be answered. FIGURE S1 Measurement procedure performed in this study [file EJN-61-0-s001.docx]

**SUPPLEMENTAL MATERIAL**

**Table A.** Reliability statistics for intra- and interday for all performed tests for the underaged group (children). Value one shows either the first test on the day and value to the second test on the day (intraday), or value 1 represents the mean within the first day and value to the mean within the second testing day (interday). The Intraclass Coefficient Correlation (ICC), Standard Error of Measurement (SEM), Minimal Detectable Change (MDC), systematic bias, Mean Absolute Error (MAE) and Mean Absolute Percentage Error (MAPE) were calculated between the two respective values, depending on the research question to be answered.

| **Test** | **Mean±SD (value 1)** | **Mean±SD (value 2)** | **ICC (95% CI)** | **SEM** | **MDC** | **Systematic bias (level of sig.)** | **MAE** | **MAPE** |
| --- | --- | --- | --- | --- | --- | --- | --- | --- |
| RulerDrop (in cm) 1_2 | 27.31±8.59 | 19.74±6.91 | 0.52 (-0.20 – 0.79) | 6.23 | 17.26 | 7.57 (<0.001) | 9.16 | 49.31 |
| RulerDrop (in cm) 2_3 | 19.74±6.91 | 15.64±7.96 | 0.73 (0.36 – 0.89) | 3.56 | 9.86 | 4.10 (0.010) | 8.61 | 68.16 |
| RulerDrop (in cm) 3_4 | 15.64±7.96 | 15.38±7.23 | 0.82 (0.56 – 0.93) | 2.54 | 7.05 | 0.26 (0.84) | 7.5 | 76.52 |
| RulerDrop (in cm) 4_5 | 15.38±7.23 | 11.42±5.36 | 0.58 (-0.01 – 0.83) | 4.80 | 13.31 | 3.95 (0.014) | 6.34 | 73.54 |
| RulerDrop (in cm) 1_5 | 27.31±8.59 | 11.42±5.36 | 0.52 (-0.15 – 0.80) | 5.64 | 15.63 | 15.89 (<0.001) | 15.89 | 198.45 |
| Trail Making Test in s 1_2 | 38.55±11.14 | 31.51±9.58 | 0.88 (0.71 – 0.95) | 2.37 | 6.57 | 7.04 (<0.001) | 7.90 | 28.36 |
| Trail Making Test in s 2_3 | 31.51±9.58 | 27.79±9.06 | 0.89 (0.74 – 0.95) | 1.95 | 5.40 | 3.71 (0.007) | 5.34 | 20.74 |
| Trail Making Test in s 3_4 | 27.79±9.06 | 20.82±7.94 | 0.93 (0.84 – 0.97) | 1.13 | 3.14 | 6.97 (<0.001) | 6.97 | 37.56 |
| Trail Making Test in s 4_5 | 20.82±7.94 | 19.44±6.51 | 0.95 (0.89 – 0.98) | 0.69 | 1.92 | 1.39 (0.05) | 2.37 | 11.74 |
| Trail Making Test in s 1_5 | 38.55±11.14 | 19.44±6.51 | 0.79 (0.50 – 0.91) | 3.46 | 9.58 | 19.11 (<0.001) | 19.11 | 107.30 |
| Stroop Test in s 1_2 | 31.36±10.05 | 29.67±9.42 | 0.88 (0.91 – 0.96) | 2.20 | 6.10 | 1.68 (0.23) | 4.50 | 15.19 |
| Stroop Test in s 2_3 | 29.67±9.42 | 26.43±7.28 | 0.90 (0.76 – 0.96) | 1.60 | 4.44 | 3.24 (0.007) | 4.07 | 15.34 |
| Stroop Test in s 3_4 | 26.43±7.28 | 24.58±5.95 | 0.97 (0.93 – 0.99) | 0.38 | 1.06 | 1.85 (0.001) | 2.11 | 8.14 |
| Stroop Test in s 4_5 | 24.58±5.95 | 24.79±5.91 | 0.99 (0.97 – 0.99) | 0.23 | 0.63 | -0.21 (0.46) | 0.95 | 4.03 |
| Stroop Test in s 1_5 | 31.36±10.05 | 24.79±5.91 | 0.89 (0.73 – 0.95) | 1.05 | 2.92 | 6.57 (<0.001) | 6.87 | 26.74 |
| Choice reaction 1_2 | 37.34±5.20 | 40.11±4.86 | 0.93 (0.83 – 0.97) | 0.68 | 1.90 | -2.77 (<0.001) | 2.86 | 7.15 |
| Choice reaction 2_3 | 46.84±6.86 | 43.09±5.28 | 0.87 (0.69 – 0.95) | 1.23 | 3.42 | -2.98 (<0.001) | 3.70 | 8.48 |
| Choice reaction 3_4 | 49.02±6.83 | 44.50±4.99 | 0.93 (0.84 – 0.97) | 0.67 | 1.86 | -1.41 (0.017) | 2.23 | 4.95 |
| Choice reaction 4_5 | 50.15±6.49 | 44.66±5.07 | 0.93 (0.84 – 0.97) | 0.68 | 1.89 | -0.16 (0.78) | 1.89 | 4.26 |
| Choice reaction 1_5 | 37.34±5.20 | 44.66±5.07 | 0.65 (0.17 – 0.86) | 3.08 | 8.54 | -7.32 (<0.001) | 7.45 | 16.30 |
| Reaction time 1_2 | 685.27±122.09 | 627.21±87.15 | 0.88 (0.72 – 0.95) | 23.78 | 65.92 | 58.07 (<0.001) | 61.98 | 9.79 |
| Reaction time 2_3 | 627.21±87.15 | 578.05±82.92 | 0.85 (0.64 – 0.94) | 23.87 | 66.17 | 49.16 (<0.001) | 64.61 | 11.37 |
| Reaction time 3_4 | 578.05±82.92 | 551.82±72.58 | 0.93 (0.84 – 0.97) | 10.25 | 28.41 | 26.23 (0.005) | 34.82 | 6.44 |
| Reaction time 4_5 | 551.82±72.58 | 551.55±72.58 | 0.92 (0.81 – 0.97) | 11.47 | 31.79 | 0.27 (0.96) | 29.45 | 5.35 |
| Reaction time 1_5 | 685.27±122.09 | 551.55±72.58 | 0.55 (-0.08 – 0.81) | 75.25 | 208.58 | 133.73 (<0.001) | 137.05 | 25.75 |

**Intraday**

| **Test** | **Mean±SD (value 1)** | **Mean±SD (value 2)** | **ICC (95% CI)** | **SEM** | **MDC** | **Systematic bias (level of sig.)** | **MAE** | **MAPE** |
| --- | --- | --- | --- | --- | --- | --- | --- | --- |
| RulerDrop 1 | 29.59±10.30 | 25.02±9.73 | 0.57 (0.03 – 0.81) | 7.35 | 20.37 | 4.57 (0.06) | 9.16 | 49.31 |
| RulerDrop 2 | 23.05±8.51 | 16.43±6.88 | 0.69 (0.29 – 0.86) | 4.30 | 11.91 | 6.61 (<0.001) | 8.61 | 68.16 |
| RulerDrop 3 | 17.46±8.37 | 13.82±9.75 | 0.63 (0.17 – 0.84) | 5.87 | 16.25 | 3.64 (0.08) | 7.5 | 76.52 |
| RulerDrop 4 | 18.57±8.52 | 12.18±7.61 | 0.69 (0.30 – 0.87) | 4.46 | 12.36 | 6.39 (<0.001) | 8.39 | 124.08 |
| RulerDrop 5 | 12.66±7.34 | 10.18±6.70 | 0.20 (-0.81 – 0.65) | 8.57 | 23.75 | 2.48 (0.22) | 7.16 | 110.32 |
| Trail Making Test 1 | 44.91±12.42 | 32.19±10.12 | 0.92 (0.81 – 0.96) | 1.83 | 5.07 | 12.71 (<0.001) | 12.77 | 42.09 |
| Trail Making Test 2 | 33.83±9.55 | 29.18±10.35 | 0.87 (0.70 – 0.94) | 2.50 | 6.93 | 4.65 (0.003) | 6.71 | 27.40 |
| Trail Making Test 3 | 30.69±10.60 | 24.89±8.28 | 0.85 (0.65 – 0.93) | 2.75 | 7.63 | 5.80 (<0.001) | 7.40 | 32.63 |
| Trail Making Test 4 | 22.05±8.89 | 19.60±7.00 | 0.94 (0.86 – 0.97) | 0.98 | 2.71 | 2.44 (0.007) | 3.31 | 16.67 |
| Trail Making Test 5 | 20.68±7.03 | 18.19±6.11 | 0.93 (0.84 – 0.97) | 0.92 | 2.56 | 2.49 0.002) | 3.19 | 18.82 |
| Stroop Test 1 | 33.22±10.65 | 29.51±9.27 | 0.97 (0.93 – 0.99) | 0.64 | 1.76 | 3.71 (<0.001) | 4.08 | 14.45 |
| Stroop Test 2 | 30.61±10.11 | 28.74±8.66 | 0.95 (0.90 – 0.98) | 0.91 | 2.51 | 1.87 (0.03) | 2.72 | 9.04 |
| Stroop Test 3 | 26.54±7.29 | 26.32±7.04 | 0.99 (0.97 – 0.99) | 0.17 | 0.48 | 0.22 (0.53) | 1.40 | 5.47 |
| Stroop Test 4 | 24.81±6.01 | 24.34±6.04 | 0.93 (0.84 – 0.97) | 0.86 | 2.37 | 0.47 (0.48) | 1.85 | 8.14 |
| Stroop Test 5 | 24.65±5.81 | 24.94±5.94 | 0.97 (0.92 – 0.99) | 0.38 | 1.06 | -0.29 (0.53) | 1.64 | 6.74 |
| Choice reaction 1 | 36.27±5.35 | 38.41±5.02 | 0.96 (0.90 – 0.98) | 0.44 | 1.21 | -2.14 (<0.001) | 2.32 | 6.07 |
| Choice reaction 2 | 40.14±4.76 | 40.09±5.35 | 0.87 (0.70 – 0.94) | 1.30 | 3.60 | 0.045 (0.95) | 2.86 | 7.62 |
| Choice reaction 3 | 43.41±4.76 | 42.77±5.76 | 0.95 (0.89 – 0.98) | 0.53 | 1.46 | 0.64 (0.20) | 1.82 | 4.36 |
| Choice reaction 4 | 44.36±4.98 | 44.64±5.11 | 0.93 (0.84 – 0.97) | 0.70 | 1.95 | -0.27 (0.62) | 2.09 | 4.83 |
| Choice reaction 5 | 44.68±5.27 | 44.64±5.14 | 0.90 (0.78 – 0.96) | 0.56 | 1.56 | 0.045 (0.95) | 2.5 | 5.63 |
| Reaction time 1 | 709.00±136.75 | 661.55±107.67 | 0.94 (0.85 – 0.97) | 15.20 | 42.13 | 47.46 (0.001) | 53.91 | 8.0 |
| Reaction time 2 | 625.09±84.77 | 629.32±107.80 | 0.79 (0.51 – 0.91) | 14.30 | 39.63 | -4.23 (0.80) | 57.5 | 8.57 |
| Reaction time 3 | 569.86±71.82 | 586.23±94.37 | 0.93 (0.84 – 0.97) | 11.72 | 32.49 | -16.36 (0.08) | 32.82 | 5.40 |
| Reaction time 4 | 555.18±71.72 | 548.46±76.17 | 0.91 (0.80 – 0.96) | 12.95 | 35.89 | 6.73 (0.45) | 33.82 | 6.08 |
| Reaction time 5 | 551.14±76.94 | 551.96±73.52 | 0.91 (0.78 – 0.96) | 13.90 | 38.53 | -0.82 (0.93) | 35.64 | 6.41 |

**Table B** shows reliability statistics for intra- and interday for all performed tests for the young adults. Value one shows either the first test on the day and value to the second test on the day (intraday), or value 1 represents the mean within the first day and value to the mean within the second testing day (interday). The Intraclass Coefficient Correlation (ICC), Standard Error of Measurement (SEM), Minimal Detectable Change (MDC), systematic bias, Mean Absolute Error (MAE) and Mean Absolute Percentage Error (MAPE) were calculated between the two respective values, depending on the research question to be answered.

| **Test** | **Mean±SD (value 1)** | **Mean±SD (value 2)** | **ICC (95% CI)** | **SEM** | **MDC** | **Systematic bias (level of sig.)** | **MAE** | **MAPE** |
| --- | --- | --- | --- | --- | --- | --- | --- | --- |
| RulerDrop (in cm) 1_2 | 13.36±3.36 | 10.71±2.89 | 0.21 (-0.90 – 0.67) | 3.70 | 10.25 | 2.65 (0.01) | 3.78 | 45.28 |
| RulerDrop (in cm) 2_3 | 10.71±2.89 | 9.48±3.16 | 0.59 (0.02 – 0.83) | 2.09 | 5.79 | 1.24 (0.11) | 2.69 | 34.11 |
| RulerDrop (in cm) 3_4 | 9.48±3.16 | 8.69±2.69 | 0.48 (-0.25 – 0.78) | 2.47 | 6.85 | 0.79 (0.32) | 2.46 | 37.61 |
| RulerDrop (in cm) 4_5 | 8.69±2.69 | 8.59±3.70 | 0.67 (0.21– 0.86) | 1.88 | 5.21 | 0.10 (0.89) | 2.65 | 44.54 |
| RulerDrop (in cm) 1_5 | 13.36±3.36 | 8.59±3.70 | 0.0 (-1 – 0.59) | 5.11 | 14.17 | 4.78 (<0.001) | 5.7 | 96.82 |
| Trail Making Test in s 1_2 | 18.74±6.17 | 15.64±3.94 | 0.61 (0.05 – 0.84) | 3.44 | 9.53 | 3.11 (0.02) | 4.80 | 31.74 |
| Trail Making Test in s 2_3 | 15.64±3.94 | 12.89±2.67 | 0.69 (0.25 – 0.87) | 1.83 | 5.07 | 2.75 (0.001) | 3.44 | 26.64 |
| Trail Making Test in s 3_4 | 12.89±2.67 | 10.11±2.71 | 0.73 (0.36 – 0.89) | 1.28 | 3.54 | 2.78 (<0.001) | 3.06 | 33.20 |
| Trail Making Test in s 4_5 | 10.11±2.71 | 10.20±2.39 | 0.94 (0.86 – 0.98) | 0.58 | 1.62 | -0.10 (0.74) | 1.02 | 10.25 |
| Trail Making Test in s 1_5 | 18.74±6.17 | 10.20±2.39 | 0.67 (0.21 – 0.86) | 0.72 | 1.98 | 8.54 (<0.001) | 8.54 | 82.98 |
| Stroop Test in s 1_2 | 18.82±2.64 | 17.68±2.26 | 0.90 (0.75 – 0.96) | 0.47 | 1.31 | 1.13 (0.003) | 1.50 | 8.63 |
| Stroop Test in s 2_3 | 17.68±2.26 | 16.83±2.26 | 0.96 (0.89 – 0.98) | 0.17 | 0.48 | 0.86 (<0.001) | 0.90 | 5.27 |
| Stroop Test in s 3_4 | 16.83±2.26 | 16.03±1.73 | 0.93 (0.89 – 0.96) | 0.24 | 0.67 | 0.80 (<0.001) | 0.88 | 5.55 |
| Stroop Test in s 4_5 | 16.03±1.73 | 15.99±1.73 | 0.89 (0.73 – 0.95) | 0.58 | 1.59 | 0.042 (0.87) | 0.90 | 5.64 |
| Stroop Test in s 1_5 | 18.82±2.64 | 15.99±1.73 | 0.77 (0.45 – 0.91) | 0.54 | 1.49 | 2.83 (<0.001) | 2.83 | 17.90 |
| Choice reaction 1_2 | 50.08±5.16 | 53.50±4.13 | 0.90 (0.77 – 0.96) | 0.88 | 2.44 | -3.43 (<0.001) | 3.83 | 7.28 |
| Choice reaction 2_3 | 53.50±4.13 | 55.48±5.25 | 0.95 (0.87 – 0.98) | 0.43 | 1.20 | -1.98 (<0.001) | 2.33 | 4.07 |
| Choice reaction 3_4 | 55.48±5.25 | 56.13±5.01 | 0.99 (0.98 – 1) | 0.10 | 0.28 | -0.65 (0.01) | 0.95 | 1.71 |
| Choice reaction 4_5 | 56.13±5.01 | 56.68±4.32 | 0.91 (0.78 – 0.96) | 1.30 | 3.59 | -0.55 (0.38) | 1.95 | 3.45 |
| Choice reaction 1_5 | 50.08±5.16 | 56.68±4.32 | 0.80 (0.51 – 0.92) | 1.22 | 3.38 | -6.6 (<0.001) | 6.6 | 11.61 |
| Reaction time 1_2 | 479.25±64.04 | 437.40±44.20 | 0.86 (0.66 – 0.94) | 14.50 | 40.20 | 41.85 (<0.001) | 47.15 | 10.56 |
| Reaction time 2_3 | 437.40±44.20 | 420.05±53.45 | 0.94 (0.85 – 0.97) | 5.82 | 16.14 | 17.35 (0.004) | 23.00 | 5.74 |
| Reaction time 3_4 | 420.05±53.45 | 411.98±46.48 | 0.98 (0.94 – 0.99) | 2.18 | 6.05 | 8.08 (0.03) | 10.63 | 2.52 |
| Reaction time 4_5 | 411.98±46.48 | 406.15±39.31 | 0.88 (0.71 – 0.95) | 9.76 | 27.05 | 5.83 (0.37) | 18.98 | 4.65 |
| Reaction time 1_5 | 479.25±64.04 | 406.15±39.31 | 0.89 (0.74 – 0.96) | 16.35 | 45.32 | 73.10 (<0.001) | 73.45 | 18.22 |

**Intraday**

| **Test** | **Mean±SD (value 1)** | **Mean±SD (value 2)** | **ICC (95% CI)** | **SEM** | **MDC** | **Systematic bias (level of sig.)** | **MAE** | **MAPE** |
| --- | --- | --- | --- | --- | --- | --- | --- | --- |
| RulerDrop 1 | 14.43±5.24 | 12.30±3.25 | 0.32 (-0.65 – 0.72) | 4.58 | 12.69 | 2.13 (0.10) | 4.83 | 42.11 |
| RulerDrop 2 | 10.55±3.12 | 10.88±3.61 | 0.66 (0.17 – 0.86) | 2.02 | 5.94 | -0.33 (0.68) | 2.88 | 58.92 |
| RulerDrop 3 | 9.25±3.28 | 9.70±3.97 | 0.68 (0.24 – 0.87) | 2.05 | 5.68 | -0.45 (0.59) | 2.85 | 35.79 |
| RulerDrop 4 | 9.30±3.73 | 8.08±3.22 | 0.32 (-0.64 – 0.72) | 3.66 | 10.14 | 1.23 (0.23) | 3.38 | 156.69 |
| RulerDrop 5 | 8.48±4.48 | 8.70±5.06 | 0.37 (-0.52 – 0.74) | 4.79 | 13.27 | -0.23 (0.87) | 4.58 | 176.77 |
| Trail Making Test 1 | 21.59±8.25 | 15.89±5.70 | 0.68 (0.23 – 0.87) | 3.23 | 8.94 | 5.70 (0.002) | 6.51 | 41.82 |
| Trail Making Test 2 | 17.09±4.69 | 14.18±3.89 | 0.80 (0.52 – 0.92) | 1.57 | 4.35 | 2.91 (0.001) | 3.55 | 25.49 |
| Trail Making Test 3 | 13.13±2.46 | 12.65±3.20 | 0.85 (0.65 – 0.94) | 0.80 | 2.20 | 0.49 (0.30) | 1.69 | 13.36 |
| Trail Making Test 4 | 10.46±3.25 | 9.76±2.49 | 0.86 (0.66 – 0.94) | 0.76 | 2.12 | 0.70 (0.14) | 1.60 | 16.05 |
| Trail Making Test 5 | 11.17±2.72 | 9.23±2.40 | 0.85 (0.65 – 0.94) | 0.71 | 1.97 | 1.94 (<0.001) | 1.98 | 23.11 |
| Stroop Test 1 | 19.52±2.97 | 18.12±2.41 | 0.95 (0.93 – 0.99) | 0.26 | 0.72 | 1.41 (<0.001) | 1.53 | 8.44 |
| Stroop Test 2 | 17.98±2.36 | 17.38±2.25 | 0.95 (0.89 – 0.98) | 0.22 | 0.60 | 0.60 (0.012) | 0.90 | 5.25 |
| Stroop Test 3 | 16.80±1.98 | 16.85±2.14 | 0.87 (0.68 – 0.94) | 0.52 | 1.45 | -0.05 (0.87) | 1.26 | 7.41 |
| Stroop Test 4 | 15.91±1.72 | 16.15±1.87 | 0.93 (0.82 – 0.97) | 0.25 | 0.70 | -0.23 (0.29) | 0.79 | 4.95 |
| Stroop Test 5 | 15.85±1.77 | 16.12±1.86 | 0.91 (0.77 – 0.96) | 0.24 | 0.66 | -0.27 (0.27) | 0.88 | 5.46 |
| Choice reaction 1 | 49.40±4.96 | 50.75±5.64 | 0.94 (0.86 – 0.98) | 0.61 | 1.70 | -1.35 (0.03) | 2.25 | 4.52 |
| Choice reaction 2 | 52.55±4.44 | 54.50±4.20 | 0.90 (0.77 – 0.96) | 0.79 | 2.19 | -1.90 (0.004) | 2.20 | 3.98 |
| Choice reaction 3 | 55.45±5.40 | 55.50±5.28 | 0.97 (0.92 – 0.99) | 0.34 | 0.94 | -0.05 (0.91) | 1.65 | 3.04 |
| Choice reaction 4 | 56.00±5.22 | 56.25±5.14 | 0.93 (0.84 – 0.97) | 0.70 | 1.93 | -0.25 (0.68) | 2.05 | 3.74 |
| Choice reaction 5 | 56.15±4.48 | 57.20±4.42 | 0.94 (0.86 – 0.98) | 0.52 | 1.44 | -1.05 (0.04) | 1.75 | 3.08 |
| Reaction time 1 | 486.35±61.06 | 472.15±71.13 | 0.93 (0.83 – 0.97) | 9.04 | 25.07 | 14.20 (0.08) | 26.90 | 5.57 |
| Reaction time 2 | 447.90±49.70 | 426.90±42.29 | 0.91 (0.78 – 0.96) | 7.95 | 22.02 | 21.00 (0.002) | 24.1 | 5.73 |
| Reaction time 3 | 420.10±54.04 | 420.00±48.85 | 0.97 (0.92 – 0.99) | 3.49 | 9.68 | 0.10 (0.98) | 16.30 | 3.76 |
| Reaction time 4 | 413.30±48.03 | 410.65±48.85 | 0.92 (0.80 – 0.97) | 7.72 | 21.40 | 2.65 (0.67) | 19.85 | 4.67 |
| Reaction time 5 | 410.95±41.67 | 401.35±39.49 | 0.93 (0.84 – 0.97) | 5.46 | 14.86 | 9.60 (0.05) | 15.5 | 3.84 |

**Table C** shows reliability statistics for intra- and interday for all performed tests for the older adults. Value one shows either the first test on the day and value to the second test on the day (intraday), or value 1 represents the mean within the first day and value to the mean within the second testing day (interday). The Intraclass Coefficient Correlation (ICC), Standard Error of Measurement (SEM), Minimal Detectable Change (MDC), systematic bias, Mean Absolute Error (MAE) and Mean Absolute Percentage Error (MAPE) were calculated between the two respective values, depending on the research question to be answered.

| **Test** | **Mean±SD (value 1)** | **Mean±SD (value 2)** | **ICC (95% CI)** | **SEM** | **MDC** | **Systematic bias (level of sig.)** | **MAE** | **MAPE** |
| --- | --- | --- | --- | --- | --- | --- | --- | --- |
| RulerDrop (in cm) 1_2 | 18.23±5.36 | 15.32±4.47 | 0.50 (0.13 – 0.75) | 3.03 | 8.40 | 2.91 (0.004) | 4.30 | 30.87 |
| RulerDrop (in cm) 2_3 | 15.32±4.47 | 12.67±4.86 | 0.48 (0.01 – 0.74) | 3.08 | 8.54 | 2.64 (0.007) | 4.16 | 42.78 |
| RulerDrop (in cm) 3_4 | 12.67±4.86 | 11.58±5.05 | 0.54 (0.18– 0.78) | 3.20 | 8.88 | 1.10 (0.28) | 3.58 | 46.79 |
| RulerDrop (in cm) 4_5 | 11.58±5.05 | 12.46±5.13 | 0.69 (0.40– 0.86) | 2.23 | 6.17 | -0.88 (0.30) | 3.49 | 28.20 |
| RulerDrop (in cm) 1_5 | 18.23±5.36 | 12.46±5.13 | 0.13 (-0.28 – 0.58) | 5.07 | 14.07 | 5.77 (<0.001) | 6.90 | 69.52 |
| Trail Making Test in s 1_2 | 25.55±9.28 | 23.00±7.63 | 0.83(0.60 – 0.93) | 2.67 | 7.40 | 2.55 (0.07) | 4.50 | 20.45 |
| Trail Making Test in s 2_3 | 23.00±7.63 | 19.36±6.02 | 0.90 (0.76 – 0.96) | 1.13 | 3.65 | 3.64 (<0.001) | 4.24 | 22.89 |
| Trail Making Test in s 3_4 | 19.36±6.02 | 16.72±6.39 | 0.88 (0.72 – 0.95) | 1.39 | 3.86 | 2.64 (0.01) | 3.51 | 22.83 |
| Trail Making Test in s 4_5 | 16.72±6.39 | 17.13±5.92 | 0.92 (0.81 – 0.97) | 0.98 | 2.71 | -0.41 (0.57) | 2.45 | 14.48 |
| Trail Making Test in s 1_5 | 25.55±9.28 | 17.13±5.92 | 0.68 (0.24 – 0.86) | 4.35 | 12.07 | 8.42(<0.001) | 8.76 | 56.56 |
| Stroop Test in s 1_2 | 19.68±3.58 | 18.44±3.39 | 0.91 (0.79 – 0.96) | 0.59 | 1.65 | 1.24 (0.007) | 1.63 | 9.52 |
| Stroop Test in s 2_3 | 18.44±3.39 | 17.77±3.83 | 0.97 (0.93 – 0.99) | 0.22 | 0.60 | 0.66 (0.018) | 1.11 | 6.65 |
| Stroop Test in s 3_4 | 17.77±3.83 | 17.15±3.94 | 0.96 (0.90 – 0.98) | 0.54 | 1.49 | 0.62 (0.069) | 1.21 | 7.33 |
| Stroop Test in s 4_5 | 17.15±3.94 | 17.15±3.43 | 0.98 (0.94 – 0.99) | 0.33 | 0.91 | 0.01 (0.984) | 0.82 | 4.14 |
| Stroop Test in s 1_5 | 19.68±3.58 | 17.15±3.43 | 0.89 (0.73 – 0.95) | 0.99 | 2.75 | 2.53 (<0.001) | 2.68 | 16.60 |
| Choice reaction 1_2 | 45.46±3.44 | 47.54±3.68 | 0.88 (0.72 – 0.95) | 0.80 | 2.22 | -2.09 (<0.001) | 2.70 | 5.58 |
| Choice reaction 2_3 | 47.54±3.68 | 49.07±3.48 | 0.95 (0.87 – 0.98) | 0.37 | 1.01 | -1.52 (<0.001) | 1.70 | 3.44 |
| Choice reaction 3_4 | 49.07±3.48 | 50.35±3.57 | 0.87 (0.68 – 94) | 0.88 | 2.43 | -1.28 (0.019) | 2.02 | 3.99 |
| Choice reaction T 4_5 | 50.35±3.57 | 50.91±3.23 | 0.94 (0.86 – 0.98) | 0.40 | 1.10 | -0.57 (0.108) | 1.09 | 2.11 |
| Choice reaction 1_5 | 45.46±3.44 | 50.91±3.23 | 0.72 (0.33 – 0.88) | 1.66 | 4.60 | -5.46 (<0.001) | 5.5 | 10.69 |
| Reaction time 1_2 | 530.63±48.09 | 502.04±50.28 | 0.88 (0.73 – 0.95) | 10.99 | 30.46 | 28.59 (<0.001) | 35.85 | 7.33 |
| Reaction time 2_3 | 502.04±50.28 | 484.54±44.10 | 0.94 (0.87 – 0.98) | 5.38 | 14.91 | 17.50 (<0.001) | 19.54 | 4.04 |
| Reaction time 3_4 | 484.54±44.10 | 468.65±43.11 | 0.86 (0.67 – 0.94) | 11.49 | 31.85 | 15.89 (0.021) | 25.41 | 5.47 |
| Reaction time 4_5 | 468.65±43.11 | 460.96±39.13 | 0.93 (0.84 – 0.97) | 5.55 | 15.39 | 7.70 (0.092) | 16.00 | 3.48 |
| Reaction time 1_5 | 530.63±48.09 | 460.96±39.13 | 0.72 (0.34 – 0.88) | 21.66 | 60.03 | 69.67 (<0.001) | 69-76 | 15.44 |

**Intraday**

| **Test** | **Mean±SD (value 1)** | **Mean±SD (value 2)** | **ICC (95% CI)** | **SEM** | **MDC** | **Systematic bias (level of sig.)** | **MAE** | **MAPE** |
| --- | --- | --- | --- | --- | --- | --- | --- | --- |
| RulerDrop 1 | 18.41±5.93 | 18.04±7.01 | 0.55 (-0.05 – 0.81) | 4.91 | 13.61 | 0.37 (0.811) | 4.89 | 28.80 |
| RulerDrop 2 | 14.41±5.75 | 16.22±4.17 | 0.74 (0.38 – 0.89) | 2.34 | 6.49 | -0.80(0.073) | 3.93 | 24.74 |
| RulerDrop 3 | 11.89±5.50 | 13.46±6.35 | 0.51 (-0.16 – 0.79) | 4.78 | 13.24 | -1.57 (0.283) | 4.91 | 55.61 |
| RulerDrop 4 | 10.54±6.11 | 12.61±5.72 | 0.62 (0.11 – 0.84) | 3.82 | 10.58 | -2.07 (0.124) | 5.23 | 58.15 |
| RulerDrop 5 | 12.15±4.95 | 12.76±7.50 | 0.48 (-0.21 – 0.79) | 5.40 | 14.95 | -0.61 (0.79) | 5.56 | 53.92 |
| Trail Making Test 1 | 29.47±11.38 | 21.63±7.77 | 0.90 (0.23 – 0.87) | 1.88 | 5.21 | 7.84 (<0.001) | 8.10 | 37.89 |
| Trail Making Test 2 | 25.31±9.15 | 20.70±6.42 | 0.93 (0.83 – 0.97) | 1.09 | 3.02 | 4.61 (<0.001) | 4.92 | 24.20 |
| Trail Making Test 3 | 21.02±6.89 | 17.70±5.77 | 0.89 (0.73 – 0.95) | 1.35 | 3.75 | 3.32 (<0.001) | 3.83 | 23.25 |
| Trail Making Test 4 | 18.13±6.85 | 15.31±6.65 | 0.89 (0.89 – 0.98) | 1.44 | 3.99 | 2.82 (0.005) | 3.73 | 23.30 |
| Trail Making Test 5 | 18.05±6.19 | 16.22±5.95 | 0.95 (0.65 – 0.94) | 0.60 | 1.67 | 1.82 (0.004) | 2.29 | 15.00 |
| Stroop Test 1 | 20.29±3.72 | 19.07±3.80 | 0.90 (0.76 – 0.96) | 0.71 | 1.99 | 1.22 (0.017) | 1.73 | 8.98 |
| Stroop Test 2 | 18.82±3.94 | 18.05±3.24 | 0.97 (0.92 – 0.99) | 0.22 | 0.60 | 0.77 (0.007) | 1.02 | 5.56 |
| Stroop Test 3 | 18.11±3.65 | 17.44±4.08 | 0.98 (0.95 – 0.99) | 0.16 | 0.45 | 0.67 (0.011) | 1.01 | 5.93 |
| Stroop Test 4 | 17.23±3.90 | 17.08±4.07 | 0.98 (0.95 – 0.99) | 0.17 | 0.47 | 0.15 (0.551) | 0.94 | 5.57 |
| Stroop Test 5 | 17.29±3.40 | 17.00±5.60 | 0.96 (0.92 – 0.99) | 0.26 | 0.72 | 0.29 (0.301) | 1.05 | 6.37 |
| Choice reaction 1 | 43.74±4.06 | 47.17±3.26 | 0.86 (0.66 – 0.94) | 0.98 | 2.70 | -3.44 (<0.001) | 3.52 | 7.49 |
| Choice reaction 2 | 46.35±4.13 | 48.74±3.53 | 0.91 (0.79 – 0.96) | 1.06 | 2.94 | -2.39 (<0.001) | 2.39 | 4.92 |
| Choice reaction 3 | 48.00±3.73 | 50.13±3.51 | 0.92 (0.80 – 0.97) | 0.60 | 1.67 | -2.13 (<0.001) | 2.48 | 4.98 |
| Choice reaction 4 | 49.83±4.55 | 50.87±2.94 | 0.85 (0.66 – 0.94) | 1.07 | 2.98 | -1.04 (0.085) | 2.00 | 4.01 |
| Choice reaction 5 | 50.7±3.71 | 51.13±2.90 | 0.94 (0.86 – 0.98) | 0.39 | 1.08 | -0.44 (0.203) | 1.30 | 2.61 |
| Reaction time 1 | 555.26±61.10 | 506.00±41.13 | 0.83 (0.59 – 0.93) | 15.87 | 43.99 | 49.26 (<0.001) | 50.39 | 9.92 |
| Reaction time 2 | 518.57±59.01 | 585.52±46.18 | 0.89 (0.74 – 0.95) | 11.17 | 30.96 | 33.04 (<0.001) | 33.57 | 6.91 |
| Reaction time 3 | 496.61±50.91 | 472.48±41.10 | 0.90 (0.76 – 0.96) | 8.85 | 24.52 | 24.13 (<0.001) | 28.83 | 6.03 |
| Reaction time 4 | 479.70±56.55 | 457.61±34.55 | 0.82 (0.57 – 0.92) | 15.58 | 43.20 | 22.09 (0.009) | 29.39 | 6.27 |
| Reaction time 5 | 463.17±45.85 | 458.74±34.19 | 0.93 (0.84 – 0.97) | 5.41 | 14.99 | 4.44 (0.309) | 16.43 | 3.50 |

**FIGURE A**. Measurement procedure performed in this study
